# Fecal Microbiota Transplantation from Toddler Donors Ameliorated DSS-Induced Colitis in Mice by Reshaping Gut Microbiota

**DOI:** 10.3390/nu18101611

**Published:** 2026-05-19

**Authors:** Yizhi Jing, Xiaoyue Bai, Yun Ji, Zhengyuan Zhai, Youyou Zhao, Yanling Hao

**Affiliations:** 1 Key Laboratory of Precision Nutrition and Food Quality, Department of Nutrition and Health, China Agricultural University, Beijing 100193, China; j15502414985@163.com; 2 College of Food Science and Nutritional Engineering, China Agricultural University, Beijing 100083, China; baixiaoyue21@163.com (X.B.); zhaizy@cau.edu.cn (Z.Z.); 3 State Key Laboratory of Animal Nutrition and Feeding, China Agricultural University, Beijing 100193, China; jean500@163.com; 4 Sirio Pharma Co., Ltd., Shantou 515000, China

**Keywords:** toddler gut microbiota, fecal microbiota transplantation, inflammatory bowel disease

## Abstract

**Background/Objectives:** Gut microbiota dysbiosis is a key driver of inflammatory bowel disease (IBD), and fecal microbiota transplantation (FMT) has emerged as a potential therapeutic strategy. In this study, we investigated the protective effects of toddler-derived FMT against colitis and elucidated the underlying mechanisms. **Methods:** Firstly, fecal microbiota from healthy toddlers was transplanted into antibiotic-pretreated mice, establishing stable colonization between days 14 and 21 post-transplantation. **Results:** In a dextran sulfate sodium-induced colitis model, FMT significantly ameliorated colitis symptoms, including reduced disease activity index and restored colon length. Toddler-derived FMT improved the intestinal barrier by preserving goblet cell density and enhancing MUC2 expression. Meanwhile, colonic inflammation was alleviated by FMT, which suppressed pro-inflammatory cytokines, reduced CD4^+^ T cell counts, and associated with downregulation of JAK/STAT-related transcripts. 16S rRNA sequencing revealed that FMT remodeled the gut microbiota by enriching beneficial genera, including *Bacteroides*, *Parabacteroides*, *Blautia*, and *Akkermansia*, which correlated positively with colon length and negatively with inflammatory markers. **Conclusions:** These findings provided a theoretical foundation that toddler-derived microbiota represents a potential donor source for FMT in IBD.

## 1. Introduction

Inflammatory bowel disease (IBD) is a chronic, relapsing gastrointestinal inflammatory disorder, which is characterized by intestinal inflammation, barrier damage, and gut microbiota dysbiosis [1]. IBD currently affects approximately 0.5% of the global population, and its incidence continues an upward trend [2,3,4]. Clinical manifestations include acute diarrhea, abdominal pain, rectal bleeding, and weight loss, which impose a substantial burden on patient quality of life [5,6]. The primary factors contributing to IBD include genetic factors, environmental factors, immune dysregulation, and gut microbiota dysbiosis [7]. Among them, gut microbiota dysbiosis is increasingly recognized as a central driver of IBD pathogenesis, characterized by the decreased abundance of beneficial microbes including *Faecalibacterium prausnitzii*, *Eubacterium* and *Akkermansia*, and the increased abundance of pro-inflammatory microbes including *Actinomyces*, *Veillonella*, and *Escherichia coli* [8,9].

Fecal microbiota transplantation (FMT) is an effective method for restoring the gut microbiota. It has shown significant efficacy in treating recurrent *Clostridioides difficile* infection and is now being explored as a potential treatment for IBD [10,11]. In a clinical study evaluating the efficacy of FMT for ulcerative colitis (UC), 24% of patients receiving single-donor FMT achieved significant improvement in intestinal symptoms, accompanied by a marked increase in fecal microbial diversity [12]. However, patients treated with multi-donor FMT exhibited a higher remission rate (32%) for UC [13]. These findings suggested that the diversity of donors may influence therapeutic outcomes. In addition, donor age also influences therapeutic response [14]. In animal studies, transplantation of gut microbiota from young (3-month-old) mice into germ-free mice resulted in more effective restoration of microbial diversity and enhanced intestinal barrier function compared with transplantation using microbiota from old (18-month-old) or aged (24-month-old) mice [15]. Furthermore, transferring young microbiota to aged mice significantly boosted ISC activity and restored tissue regeneration capacity [16]. These findings highlight that donor characteristics such as age and diversity critically influence the therapeutic efficacy of FMT.

The gut microbiota of healthy toddlers is distinct from that of healthy adults [17,18]. The toddler gut microbiota is dominated by *Bifidobacterium*, accounting for approximately 90% of the microbial community [19]. *Bifidobacterium* metabolizes human milk oligosaccharides and produces immunomodulatory metabolites, such as short-chain fatty acids (SCFAs), which contribute to alleviating gastrointestinal symptoms and regulating immune function [19,20,21]. In addition, the relative abundance of *Parabacteroides* gradually increases throughout toddler development, ultimately reaching 1.27% [22]. Within this genus, *Parabacteroides distasonis*, *Parabacteroides goldsteinii*, and *Parabacteroides merdae* exert potent anti-inflammatory effects [23]. It is noteworthy that the healthy gut microbiota established during the toddler years plays a crucial role in maintaining intestinal homeostasis including enhancing epithelial integrity and promoting mucus layer development [24]. However, the transplantation of toddler-derived gut microbiota as a preventive intervention for IBD remains largely unexplored.

In this study, a mixture of fecal samples from 10 toddlers was transplanted into mice, and a stable colonization window was identified between 14 and 21 days. During the stable colonization period, we evaluated the effects of FMT pretreatment on histopathology, intestinal barrier function, inflammatory response, and microbial composition in dextran sulfate sodium (DSS)-induced colitis mice. This study investigated the protective role of toddler-derived FMT in IBD colitis.

## 2. Material and Methods

### 2.1. Toddler Fecal Sample Collection

Fecal samples were collected from 10 healthy toddler donors (aged 12–36 months) residing in Beijing, China. The toddlers had not received antibiotics or probiotics for six months before enrolling. The specific inclusion and exclusion criteria for donors are detailed in Supplementary Table S1. All donors tested negative for specific pathogens, including viruses, Mycoplasma pneumoniae, and parasites. Fecal samples from the 10 donors were collected under anaerobic conditions and mechanically homogenized. The homogenized feces were then dispersed in a sterile saline and glycerol mixture at a 10% (*w*/*v*) ratio. After preliminary filtration using sterile gauze, the suspension was further filtered through a sterile 100-μm filter to prepare the fecal bacterial suspension. Fecal samples from the 10 donors were pooled prior to transplantation. The final fecal suspension had an estimated bacterial load of approximately 1 × 10^9^ CFU/mL, as determined by anaerobic plating on BHI agar. Pooling was performed to minimize donor-to-donor variability and to provide a standardized inoculum. All participating mothers provided written informed consent. The study was approved by the Ethics Committee of Human Research at China Agricultural University (approval number: CAUHR-20240701).

### 2.2. 16S rRNA Gene Sequencing and Analysis

Total genomic DNA was extracted from fecal samples. Then, the V3-V4 hypervariable region of the bacterial 16S rRNA gene was amplified using the universal primer pair 338F and 806R. Amplicons were subsequently sequenced on the Illumina MiSeq platform. Bioinformatics analysis was performed on the MajorBioCloud Platform (www.MajorBio.com). Briefly, raw paired-end reads were analyzed using QIIME 263, followed by taxonomic assignment using the RDP Classifier against the SILVA 138 reference database. Principal coordinate analysis (PCoA) was based on Bray–Curtis distances. Differentially enriched taxa were identified using the Kruskal–Wallis test followed by Dunn’s post hoc test. Correlation analysis was performed using Spearman’s rank correlation coefficient and linear regression. The raw sequencing data have been deposited in the National Center for Biotechnology Information (NCBI) database; the 16S rRNA gene sequencing accession number is PRJNA1367683 (https://www.ncbi.nlm.nih.gov/bioproject/PRJNA1367683, accessed on 21 November 2025).

### 2.3. Animal Experiments

All animal experiments were approved by the Animal Ethics Committee of China Agricultural University (AW31604202-4-1). 5-week-old male C57BL/6J mice (Charles River Laboratories, Beijing, China) were housed under specific-pathogen-free (SPF) conditions (12 h light/dark cycle, 23 ± 1 °C, 55 ± 15% humidity) with free access to food and water. Before the experiment, all mice were adapted for 7 days.

To evaluate the colonization potential of toddler gut microbiota, 12 mice were randomly divided into two groups (*n* = 6): the control group and the FMT group. The FMT group was pretreated with a 7-day administration of a four-antibiotic cocktail (ABX) including ampicillin sodium (200 mg/kg body weight; Macklin, Beijing, China), neomycin sulfate (200 mg/kg body weight; Biotopped, Beijing, China), metronidazole (200 mg/kg body weight; Macklin), and vancomycin hydrochloride (100 mg/kg body weight; Macklin). Then, fecal microbial suspension (200 µL) via oral gavage was carried out for three days. During this period, the control group received an equal volume of sterile phosphate-buffered saline (PBS) via oral gavage. Fecal samples from the FMT group were respectively collected at post-antibiotic treatment, days 7, 14, and 21 post-FMT. Additionally, fecal samples from the control group were collected as a baseline reference.

To evaluate the effects of toddler gut microbiota on IBD colitis, 18 mice were randomly divided into three groups (*n* = 6): the Control group, the DSS group, and the FMTDSS group. From day 1 to day 7, the FMTDSS group received a ABX pretreatment, followed by daily oral gavage of 200 µL fecal microbiota suspension on days 8–10. Meanwhile, the Control and DSS groups were given an equivalent volume of PBS via oral gavage days 1 to day 10. From day 24 to day 31, mice in the DSS and FMTDSS groups were provided with drinking water containing 2.5% (*w*/*v*) DSS (MP Biologicals, Santa Ana, CA, USA), whereas the Control group received normal drinking water. Freshly fecal samples were collected on day 31, snap-frozen and stored at −80 °C. All 30 mice were then anesthetized with isoflurane and euthanized, and blood samples were collected via retro-orbital puncture. The colon was dissected, and its contents were collected. Colonic tissue was fixed in 4% paraformaldehyde for histology or snap-frozen and stored at −80 °C for subsequent analyses.

### 2.4. Disease Activity Index and Histological Analysis

During the DSS treatment period, the Disease Activity Index (DAI) score was assessed daily based on body weight loss, stool consistency, and fecal occult blood (Table S2). Following dissection, colon tissues were fixed in 4% paraformaldehyde at room temperature for 24 h. After fixation, the tissues were embedded, sectioned, and stained with hematoxylin and eosin (H&E) (Table S3). Additionally, Alcian blue-periodic acid Schiff (AB-PAS) staining was performed to evaluate goblet cell distribution. Histological observations were conducted under a light microscope (Leica Microsystems, Wetzlar, Germany), and images were captured for further analysis. Histological scoring and all image quantifications were performed by two independent observers blinded to the experimental groups.

### 2.5. Quantitative Reverse Transcription PCR

Total RNA was extracted using AG RNAex Pro reagent (Accurate Biotechnology Hunan Co., Ltd., Changsha, China) according to the manufacturer’s instructions. Reverse transcription was performed using Evo M-MLV RT Mix Kit with gDNA Clean (Accurate Biotechnology), and qPCR was conducted using SYBR Green Premix Pro Taq Hs qPCR kit (Accurate Biotechnology) on a QuantStudio™5 Real-Time PCR System (Thermo Fisher, Wilmington, NC, USA). Primers for RT-qPCR were synthesized by Sangon Biotech (Shanghai) Co., Ltd. (Shanghai, China) and were listed in Table S4. The relative gene expressions were calculated using the 2^−ΔΔCt^ method using *β-actin* as the reference gene [25].

### 2.6. Immunofluorescence Staining

Colonic sections were blocked with blocking buffer (Beyotime Biotech, Beijing, China), followed by incubation with primary antibodies MUC2 (Proteintech, 27675-1-AP) and CD4^+^ (Santa Cruz, SC-19641) for 12 h at 4 °C. Sections were then incubated with an Alexa Fluor 488-conjugated secondary antibody (Abcam, ab150077) for 1 h at 25 °C. Nuclei were counterstained with DAPI (C1005, Beyotime, Shanghai, China). Fluorescent images were captured using a fluorescence microscope (DM6B; Leica Microsystems, Germany). Five random fields per section were examined for each mouse. All samples were captured using identical acquisition settings (exposure time, gain, offset). Background fluorescence was subtracted based on a region without primary antibody staining, and quantification was carried out by two independent observers blinded to group allocation.

### 2.7. Statistical Analysis

Statistical analysis was performed using GraphPad Prism 9.5 (GraphPad Software, San Diego, CA, USA). The Shapiro–Wilk test was performed to assess the normality of each data set. Data are presented as mean ± standard error of the mean (SEM). Statistical comparisons among groups were performed using one-way analysis of variance (ANOVA) followed by Tukey’s multiple comparison test. For microbiome differential abundance analysis, the Kruskal–Wallis test followed by Dunn’s post hoc test with Benjamini–Hochberg false discovery rate (FDR) correction was applied. *p* < 0.05 was considered statistically significant.

## 3. Results

### 3.1. Toddler-Derived FMT Reshaped the Mouse Gut Microbiota Composition

**Figure 1:**
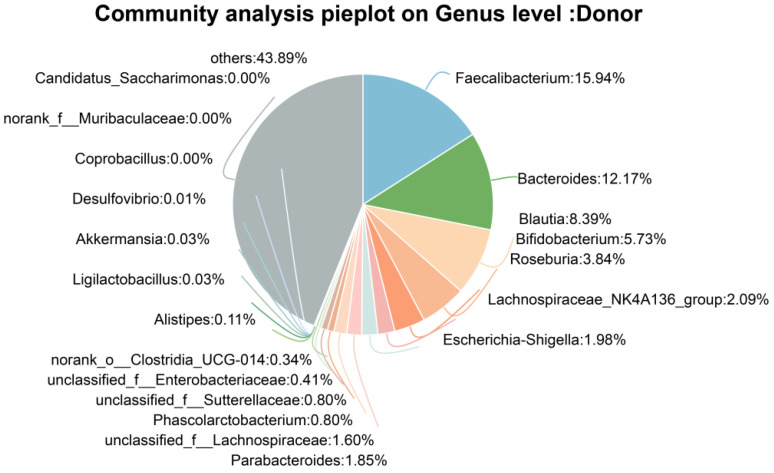
Pie chart showing the taxonomic composition of the donor gut microbiota at the genus level.

The fecal microbiota from healthy toddlers was isolated and subjected to 16S rRNA sequencing. The results showed that the donor microbiota mainly comprised 15.94% *Faecalibacterium*, 12.17% *Bacteroides*, 8.39% *Blautia*, 5.73% *Bifidobacterium*, 3.84% *Roseburia*, and 1.85% *Parabacteroides* (Figure 1). This compositional feature is consistent with the typical toddler gut microbiota, and suitable for subsequent experiments.

**Figure 2:**
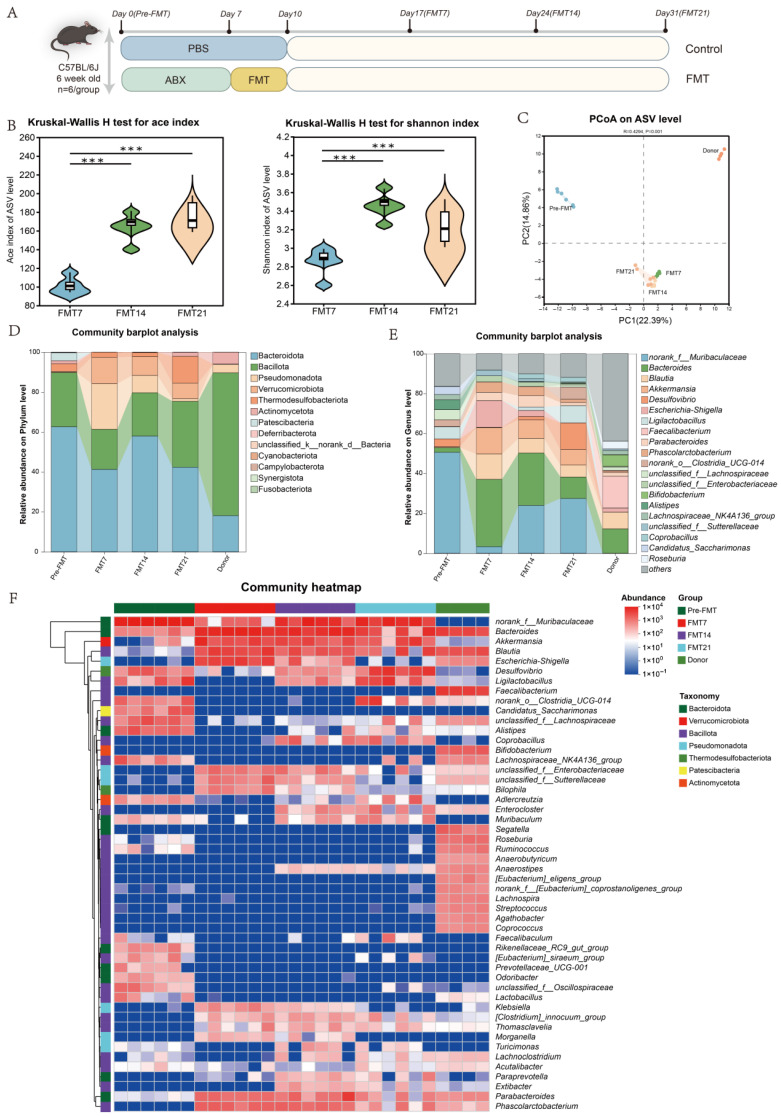
Toddler fecal microbiota transplantation reshapes the gut microbiota of mice. (**A**) Experimental design. (**B**) α-Diversity indices (Ace and Shannon). (**C**) β-Diversity based on principal co-ordinates analysis. (**D**) Relative abundance of bacteria at the phylum level. (**E**) Relative abundance of bacteria at the genus level. (**F**) Heatmap showing the genus-level microbial composition across groups. Box colors indicated the level of correlation coefficients. (*n* = 6 per group, *** *p* < 0.001).

The experimental design for evaluating the colonization potential of toddler gut microbiota is illustrated in Figure 2A. On days 7, 14, and 21 post-FMT, fecal samples were collected from recipient mice and analyzed by 16S rRNA sequencing. Compared with day 7 post-FMT, the ACE and Shannon indices were significantly higher on day 14, and this increase was maintained through day 21 (Figure 2B). Beta-diversity analysis showed that the fecal samples from the pre-FMT, donor, and post-FMT groups formed separate clusters (Figure 2C). Among the first principal coordinate (PC1), the post-FMT group was closer to the donor microbiota than the pre-FMT group. At the phylum level, Bacteroidota and Bacillota predominated in all groups (Figure 2D). Similar to the donor group, the FMT mice also showed an increase in Pseudomonadota. The microbial composition at the genus level is presented in Figure 2E. The pre-FMT group microbiota was dominated by *norank_f__Muribaculaceae*, *Desulfovibrio*, *Ligilactobacillus*, norank_o__Clostridia_UCG-014, *Alistipes*, and *Candidatus*_Saccharimonas. The donor gut microbiota primarily consisted of *Bacteroides*, *Blautia*, *Faecalibacterium*, *Parabacteroides*, *Phascolarctobacterium*, *Bifidobacterium*, and *Coprobacillus*. After FMT, the mouse gut microbiota consisted mainly of *norank_f__Muribaculaceae*, *Bacteroides*, *Blautia*, *Parabacteroides*, and *Phascolarctobacterium.* In addition, genus-level heatmap analysis revealed that the microbial composition on days 7, 14, and 21 post-FMT continued to shift over time (Figure 2F). Specifically, several genera were already detected in the mouse gut by day 7 post-FMT, including *Phascolarctobacterium*, *Parabacteroides*, *Bilophila*, *Thomasclavelia*, *Acutalibacter*, and *Blautia*. At day 14 post-FMT, the other genera including *Enterobacter*, *Anaerostipes*, *Lachnoclostridium*, and *Extibacter* were identified in the mouse gut and remained present through day 21 post-FMT.

**Figure 3:**
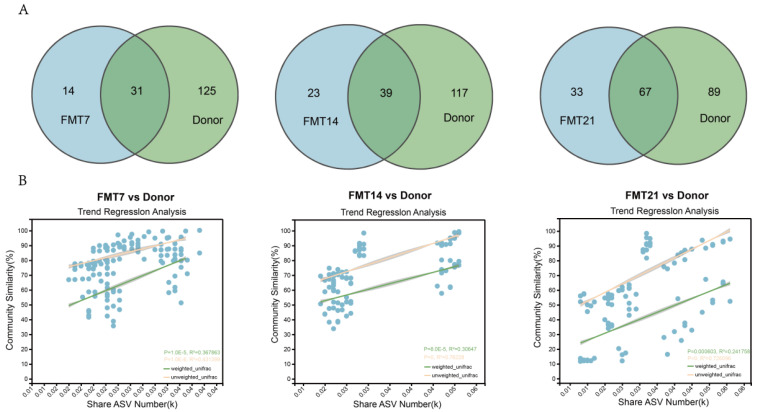
The stable colonization window of the toddler-derived gut microbiota after FMT. (**A**) Venn diagram of shared and unique gut bacterial genera. (**B**) Convergent species-level community similarity patterns inferred from weighted and unweighted UniFrac distance matrices.

Venn diagram analysis at the genus level revealed a continuous increase in the number of shared bacterial genera between the recipient mouse gut microbiota and the donor microbiota (Figure 3A). Specifically, 31, 39, and 67 common genera were identified between the donor and recipient on days 7, 14, and 21 post-FMT. Linear regression analysis was performed to evaluate the relationship between the number of shared ASVs and beta diversity distances among samples (Figure 3B). On day 7 post-FMT, 0.04k ASVs were shared between recipient mice and the donor microbiota, with an R^2^ value of 0.43 based on the unweighted UniFrac distance matrix. On day 14 post-FMT, the number of shared ASVs increased to 0.06k (R^2^ = 0.73), and this level persisted until day 21 (R^2^ = 0.76). These findings demonstrated that toddler-derived FMT reshaped the mouse gut microbiota composition and maintained high similarity to the donor microbiota from days 14 to 21 post-transplantation.

### 3.2. Toddler-Derived FMT Alleviated DSS-Induced Colitis in Mice

**Figure 4:**
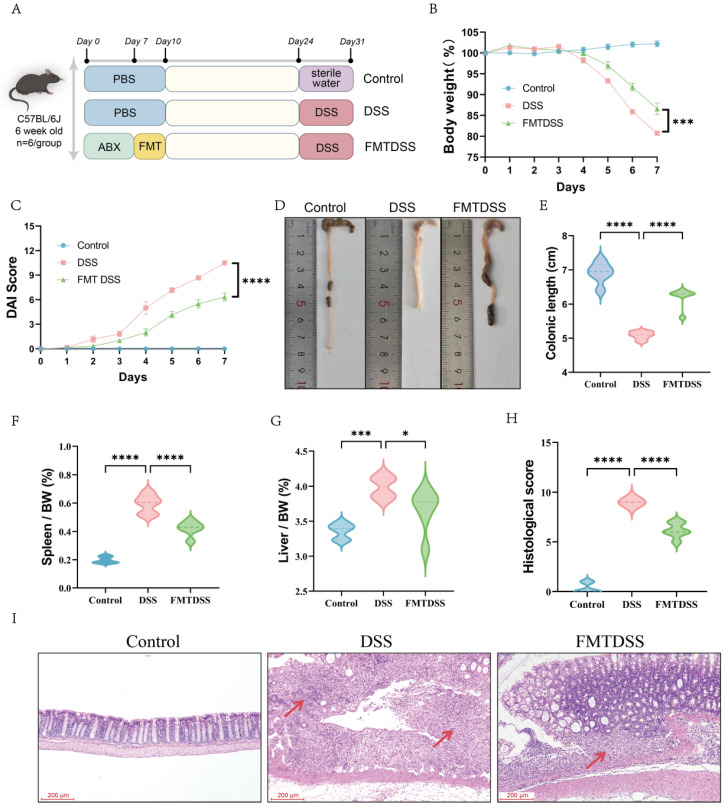
FMT ameliorates colitis in mice. (**A**) Experimental design. (**B**) Body weight changes. (**C**) DAI score. (**D**) Representative colon images. (**E**) Colonic length. (**F**) Spleen index. (**G**) Liver index. (**H**) Histological score. (**I**) Representative H&E staining of colon sections in each group (scale bar, 200 µm). Red arrows indicate inflammatory cell infiltration and crypt disruption. (*n* = 6 per group, * *p* < 0.05, *** *p* < 0.001, and **** *p* < 0.0001).

The experimental design for evaluating the effects of the toddler gut microbiota on IBD colitis is illustrated in Figure 4A. DSS treatment significantly reduced body weight and increased DAI score compared to controls, whereas the FMTDSS group showed higher body weight and lower DAI scores than the DSS group (Figure 4B,C). Furthermore, the colon length was markedly decreased in the DSS group (5.1 cm) compared with the control group (6.9 cm), while FMT restored the colon length content to 6.3 cm (Figure 4D,E). Additionally, FMT reduced the DSS-induced increases in spleen and liver indices, suggesting attenuation of systemic inflammatory responses (Figure 4F,G). H&E staining revealed extensive epithelial damage, crypt destruction, and pronounced inflammatory cell infiltration in the colonic tissue of the DSS group (Figure 4H). However, these pathological alterations were markedly alleviated in the FMTDSS group, which exhibited lower histological scores compared to the DSS group (Figure 4I). These findings demonstrated that transplantation of toddler gut microbiota mitigated DSS-induced colitis.

### 3.3. Toddler-Derived FMT Ameliorated Intestinal Barrier Dysfunction

**Figure 5:**
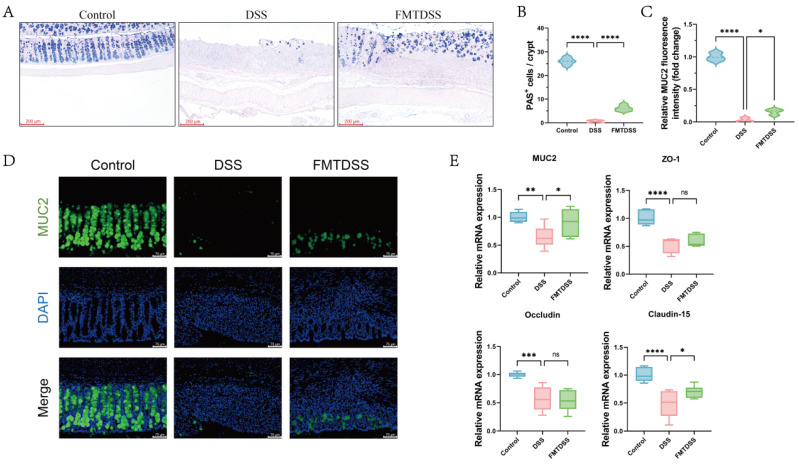
FMT improves intestinal barrier. (**A**) Representative AB-PAS staining of colon sections in each group (scale bar = 200 µm). (**B**) Quantification of goblet cells per crypt. (**C**) Quantification of the MUC2 fluorescence intensity. (**D**) Representative immunofluorescence staining of MUC2 (green), with nuclei counterstained with DAPI (blue) (scale bar = 75 μm). (**E**) The mRNA levels of *MUC2*, *ZO-1*, *Occludin*, and *Claudin-15* in colon tissues. (*n* = 6 per group, * *p* < 0.05, ** *p* < 0.01, *** *p* < 0.001, and **** *p* < 0.0001, ns indicates not significant).

AB-PAS staining revealed that goblet cells were almost completely absent in the crypts of the DSS group (Figure 5A), but FMT significantly increased the number of goblet cells per crypt (Figure 5B). Immunofluorescence staining further confirmed reduced expression of MUC2 in the DSS group, whereas FMT significantly restored their expression toward control levels (Figure 5C,D). Furthermore, compared with the control group, the mRNA expression levels of *MUC2*, *ZO-1*, *Occludin*, and *Claudin-15* were significantly downregulated in the DSS group (Figure 5E). Moreover, FMT upregulated the transcript levels of *MUC2* and *Claudin-15* by 1.40- and 1.45-fold compared with the DSS group. These results indicated that FMT can mitigate DSS-induced depletion of colonic goblet cells and disruption of the intestinal mechanical barrier.

### 3.4. Toddler-Derived FMT Ameliorated Intestinal Inflammation

**Figure 6:**
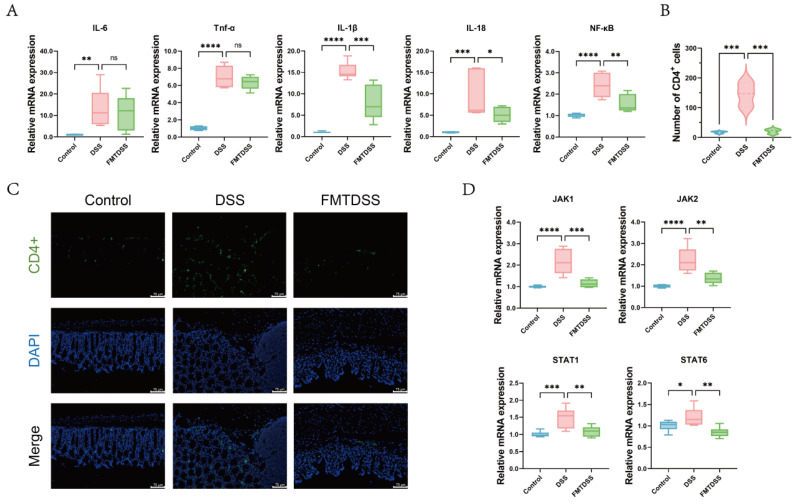
FMT promotes intestinal health by suppressing inflammatory response. (**A**) The mRNA levels of *IL-6*, *Tnf-α*, *IL-1β*, *IL-18* and *NF-κB* in colon tissues. (**B**) Quantification of CD4^+^ cells. (**C**) Representative immunofluorescence staining of CD4^+^ cells (green), with nuclei counterstained with DAPI (blue) (scale bar = 75 μm). (**D**) The mRNA levels of *JAK1*, *JAK2*, *STAT1*, and *STAT6* in colon tissues. (*n* = 6 per group, * *p* < 0.05, ** *p* < 0.01, *** *p* < 0.001, and **** *p* < 0.0001, ns indicates not significant).

To evaluate the effect of FMT on colonic inflammation, the expression levels of inflammation-related genes were examined in mice colonic tissues. The mRNA expression levels of pro-inflammatory cytokines, including *IL-6*, *Tnf-α*, *IL-1β*, and *IL-18*, as well as *NF-κB*, were significantly elevated in the DSS group (Figure 6A). In contrast, FMT treatment markedly reduced the expression of *IL-1β*, *IL-18*, and *NF-κB*, indicating that FMT alleviates low-grade colonic inflammation. In addition, FMT decreased DSS-induced CD4^+^ T cell accumulation in colonic tissues (Figure 6B,C). Furthermore, compared with the DSS group, the mRNA expression levels of *JAK1*, *JAK2*, *STAT1*, and *STAT6* were significantly decreased in the FMTDSS group (Figure 6D). These results demonstrated that FMT alleviated DSS-induced colitis by reducing pro-inflammatory cytokine expression and suppressing excessive immune activation within the colonic mucosa.

### 3.5. Toddler-Derived FMT Modulates the Gut Microbiota Composition

**Figure 7:**
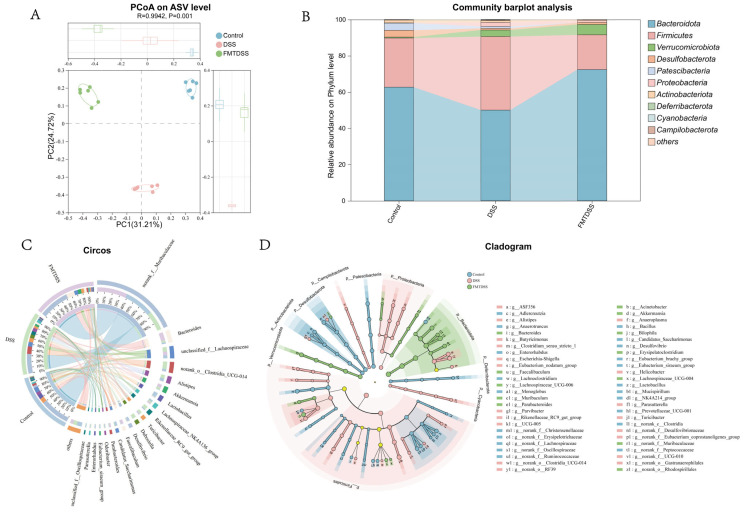
Effects of FMT on gut microbiota composition in DSS-induced colitis mice. (**A**) β-Diversity based on principal co-ordinates analysis. (**B**) Relative abundance of bacteria at the phylum level. (**C**) Circos plot of dominant gut bacterial genera. (**D**) LEfSe analysis showing the enriched bacterial taxa (phylum to genus level) in each group. (*n* = 6 per group).

To evaluate the role of the gut microbiota in the anti-colitis effect of FMT, 16S rRNA sequencing was performed on mice feces. PCoA showed distinct clustering of microbial communities across three groups (Figure 7A). At the phylum level, FMT increased the relative abundance of Bacteroidota and Firmicutes compared with the DSS group (Figure 7B). At the genus level, the control group was mainly enriched with *norank_f__Muribaculaceae* and *Lactobacillus* (Figure 7C). The DSS group was predominantly enriched with *norank_o__Clostridia_UCG-014*, *Alistipes*, *Rikenellaceae_RC9_gut_group*, and *Dubosiella*. In contrast, the FMTDSS group was primarily enriched with *norank_f__Muribaculaceae*, *Bacteroides*, *Akkermansia*, and *Faecalibaculum*. LEfSe analysis revealed that the DSS group showed enrichment of inflammation-associated taxa, such as *Escherichia-Shigella*, *Helicobacter*, *Butyricimonas* and *Proteobacteria* (Figure 7D). Notably, the FMTDSS group was characterized by enrichment of beneficial genera, including *Akkermansia*, *Parabacteroides*, and *Faecalibaculum*.

### 3.6. FMT Modulates Key Microbial Taxa Associated with Colitis-Related Phenotypes

**Figure 8:**
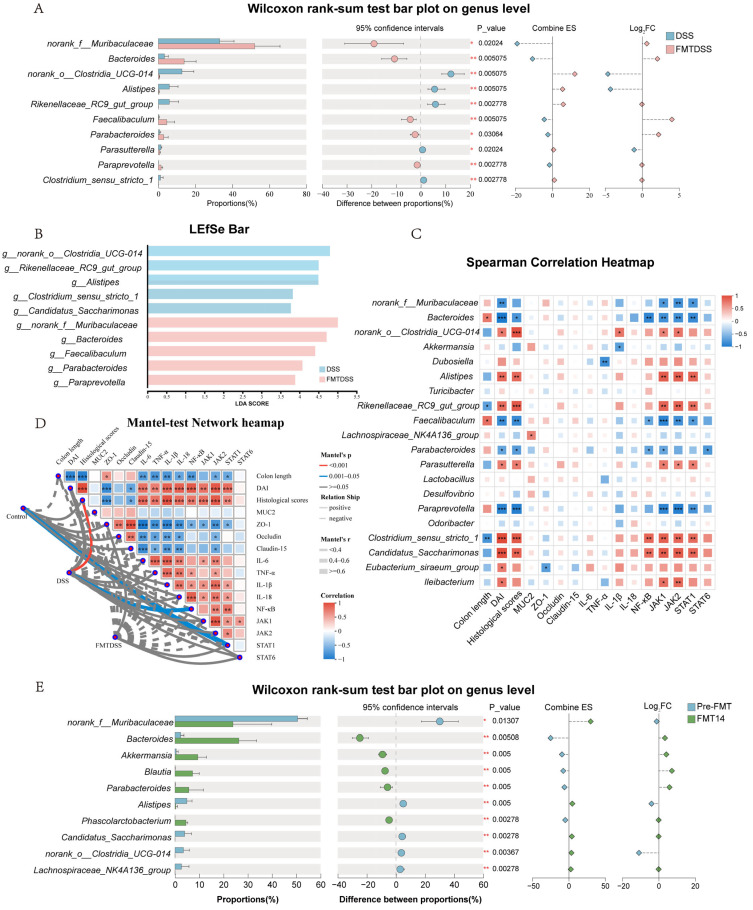
Gut microbiota composition, differential abundance, and correlation with disease phenotypes. (**A**) Wilcoxon rank-sum test bar plot on genus level of DSS-treated mice with or without FMT. (**B**) LDA scores of microbial taxa enriched in DSS and FMTDSS groups as identified by LEfSe analysis (LDA > 3.5). (**C**) Heatmap of the correlation between bacteria at the genus level and various biochemical parameters. (**D**) Mantel Test network heatmap analysis. Box colors indicated the level of correlation coefficients. (**E**) Wilcoxon rank-sum test bar plot on genus level in pre-FMT and FMT14 groups. (*n* = 6 per group, * *p* < 0.05, ** *p* < 0.01 and *** *p* < 0.001).

Microbial difference analysis revealed that the FMTDSS group exhibited decreased abundance of *norank_o_Clostridia_UCG-014*, *Alistipes*, *Rikenellaceae_RC9_gut_group*, *Parasutterella*, and *Clostridium_sensu_stricto_1* (Figure 8A). Meanwhile, the relative abundance of *norank_f__Muribaculaceae*, *Bacteroides*, *Faecalibaculum*, *Parabacteroides*, and *Paraprevotella* was significantly increased in the FMTDSS group, all of which showed higher LDA scores in the FMTDSS group (Figure 8B). Spearman’s correlation analysis revealed that *Bacteroides*, *Faecalibaculum*, *Parabacteroides*, and *Paraprevotella* were positively correlated with colon length, and negatively correlated with DAI scores, histological scores, and pro-inflammatory gene expression (Figure 8C). In contrast, *norank_o__Clostridia_UCG-014*, *Rikenellaceae_RC9_gut_group*, and *Clostridium_sensu_stricto_1* were positively correlated with DAI scores, histological scores, and pro-inflammatory gene expression. Mantel test analysis revealed that the gut microbiota was closely correlated with histological scores in the DSS group (Figure 8D). Histological scores were positively correlated with DAI scores and pro-inflammatory gene expression and negatively correlated with colon length and barrier-related gene expression. Additionally, the differential analysis between the FMT14 and pre-FMT groups at the genus level revealed that the abundances of *Bacteroides*, *Akkermansia*, *Blautia*, *Parabacteroides*, and *Phascolarctobacterium* were significantly increased on day 14 post-FMT (Figure 8E). These findings indicated that FMT protects against colitis by remodeling the gut microbiota in mice.

## 4. Discussion

Gut microbiota dysbiosis is associated with the development of various diseases, particularly intestinal disorders [26]. FMT is commonly used to investigate the relationship between gut microbe dysbiosis and disease [27]. In such studies, it is critical that donor-derived microbiota successfully colonizes the recipient gut and maintains long-term stability [28]. The colonization rate of transferring human-derived gut microbiota into germ-free or antibiotic-pretreated mice reached approximately 60% [29]. However, the colonization efficacy varies among different microbial taxa. Even closely related species exhibited markedly different colonization capacities. For instance, *Prevotella copri* and *Bacteroides vulgatus* showed relatively high engraftment rates, whereas *Prevotella tannerae* and *Bacteroides pectinophilus* exhibited poor colonization efficiency after FMT [30]. In addition, three weeks after FMT from infant donors into antibiotic-pretreated mice, donor-derived *Bacteroides*, *Parabacteroides*, and *Phascolarctobacterium* successfully colonized the mouse gut, whereas the infant-associated *Bifidobacterium* was not established [31]. In this study, the recipient mouse gut microbiota was composed primarily of *Bacteroides*, *Blautia*, *Parabacteroides*, and *Phascolarctobacterium*, whereas *Bifidobacterium* was also not detected. This phenomenon may be attributed to the adaptation of *Bifidobacterium* to the host gut and the conditions of the donor intestine [32]. *B. longum* subsp. *infantis* requires human milk oligosaccharides for successful colonization. Consequently, the observed protective effects cannot be attributed to *Bifidobacterium*. Instead, we ascribe them to the successful engraftment of other toddler-derived genera including *Bacteroides*, *Parabacteroides*, and *Blautia*. Additionally, the time required for stable colonization after FMT is donor dependent. When panda gut microbiota was transplanted into mice, the microbiota from the shoot-eating season was already stably established by day 7 after FMT, whereas that from the leaf-eating season required 14 days for stable colonization [33]. In this study, the stable colonization window of the toddler-derived gut microbiota was identified as days 14 to 21 post-FMT in mice. Therefore, the protective effect of toddler gut microbiota against DSS-induced colitis was evaluated during this window period.

Patients with IBD are primarily characterized by intestinal barrier damage and intestinal inflammation [34]. Among these, dysregulation of mucus barrier and goblet cell development significantly increase disease susceptibility [35]. Fecal microbiota from young (10-week-old) and aged (78-week-old) mice were separately transplanted into recipient mice, and the recipients of young mouse microbiota exhibited a higher number of goblet cells and lower intestinal permeability [36]. Transplanting healthy mouse fecal microbiota into septic mice improved the mucus barrier and reduced intestinal permeability by upregulating MUC2 expression, further confirming the protective effect of FMT on the intestinal mucus barrier [37]. In this study, toddler-derived FMT restored DSS-induced goblet cell reduction and upregulated MUC2 expression, thereby effectively ameliorating colonic barrier damage. In addition, in IBD patients, the abundance of CD4^+^ T cells in the mucosal epithelium is significantly increased [38]. Increased CD4^+^ T cells both activate the JAK/STAT signaling pathway to exacerbate colonic inflammation and promote the accumulation of pro-inflammatory cytokines including IL-6, IL-1β, and TNF-α [39,40]. FMT from healthy mice into colitic mice suppressed the DSS-induced increase in CD4^+^ and CD8^+^ T cells by remodeling the gut microbiota [41]. Immune cells including CD4^+^ T cells and invariant natural killer T cells are induced by FMT to produce IL-10 [42]. Furthermore, transplanting microbiota from healthy donors improved colitis in recipient mice, which was associated with decreased expression of IL-1β, IL-6 and TNF-α [43]. In this study, toddler-derived FMT also reduced the number of CD4^+^ T cells and suppressed the production of pro-inflammatory cytokines, including IL-1β and IL-18. These findings demonstrated that toddler-derived FMT alleviated colitis by restoring the intestinal mucus barrier, reducing CD4^+^ T cell infiltration, and regulating the JAK/STAT-mediated pro-inflammatory response.

FMT protects against intestinal inflammation and barrier damage by means of specific beneficial microbial taxa [44]. FMT from donors rich in *Bacteroides thetaiotaomicron* and *Faecalibacterium prausnitzii* significantly ameliorates DSS-induced colitis in mice [45]. In a mouse model of sepsis, enrichment of the Lachnospiraceae following FMT played a key role in protecting the intestinal barrier [37]. In this study, the abundances of *Bacteroides*, *Parabacteroides*, *Blautia*, and *Akkermansia* were increased in the mouse gut following FMT. These genera have been demonstrated to be closely associated with intestinal barrier function and the regulation of inflammation [46]. In particular, *Bacteroides thetaiotaomicron* and *Blautia coccoides* produce SCFAs, which were associated with goblet cell differentiation and mucin synthesis [47,48]. SCFAs are key microbial metabolites that support colonocyte energy metabolism, strengthen tight-junction integrity, and exert anti-inflammatory effects [49]. *Parabacteroides distasonis* may be linked to improved TNBS-induced colitis by regulating immunity and enhancing intestinal barrier function [50]. *Akkermansia* were correlated with enhanced mucin production and goblet cell differentiation by restoring microbiota balance [51]. In addition, polysaccharide A from *Bacteroides fragilis* mediates the conversion of CD4^+^ T cells [52]. *Parabacteroides goldsteinii* and *Blautia producta* inhibit NF-κB activation and reduce the production of pro-inflammatory cytokines [53,54]. In summary, toddler-derived FMT effectively enhances intestinal barrier function and suppresses inflammatory responses in mice by enriching multiple beneficial bacterial genera.

In addition, several limitations of this study should be acknowledged. In the present study, toddler-derived FMT was administered two weeks prior to DSS challenge, representing a preventive intervention rather than a therapeutic intervention. Therefore, the translational implications of our findings are currently limited to preventive contexts. Pooling fecal samples from 10 toddler donors reduces variability but masks inter-individual differences and may dilute key taxa, underscoring the need for future individual donor FMT comparisons. The absence of an ABX + DSS control group limits the ability to distinguish the effect of microbiota depletion from that of FMT. However, antibiotic pretreatment alone is known to worsen DSS colitis [55,56]. And our 14-day washout period eliminates direct antibiotic effects, and the protective time window aligns precisely with stable donor microbiota engraftment (Figure 3). Thus, the observed benefit is likely due to FMT, although the absence of an ABX + DSS control group means we cannot completely exclude a contributory role of antibiotic-induced depletion. No formal power analysis was performed for sample size, and that microbial sequencing was not conducted during the DSS challenge period. Nonetheless, the significant differences observed for key endpoints and the stable establishment of donor microbiota before DSS induction (Figure 3) support the robustness of our conclusions. The short-chain fatty acids (SCFAs) or other microbiota-derived metabolites were not quantified in this study. Thus, the functional contribution of enriched genera (e.g., *Bacteroides*, *Blautia*) to barrier protection and immune modulation remains inferential and requires future metabolomic validation. The correlation analyses and LEfSe results are based on a modest sample size and should be considered exploratory. In addition, the expression of tight junction proteins (ZO-1, Occludin, and Claudin-15) and JAK/STAT protein was evaluated only at the mRNA level. Future studies should include protein-level analyses to further elucidate the functional role of toddler-derived FMT in intestinal protection. Exclusive use of male mice limits generalizability, and future studies should include both sexes to assess sex-dependent protective effects of toddler-derived FMT.

## 5. Conclusions

This study demonstrates that toddler-derived FMT effectively protects against DSS-induced colitis in mice by remodeling the gut microbiota composition. Specifically, FMT increases goblet cell numbers and upregulates MUC2 expression, thereby enhancing intestinal barrier function. In addition, it reduces the number of CD4^+^ T cells and decreases the expression of pro-inflammatory cytokines, thereby ameliorating intestinal inflammation. These effects are attributed to the ability of FMT to reshape gut microbiota, which enriches multiple toddler-derived key functional genera, including *Bacteroides*, *Parabacteroides*, *Blautia*, and *Akkermansia*. Collectively, these findings indicate that toddler-derived microbiota represents a potential donor source for FMT intervention in IBD.

## Data Availability

The original contributions presented in this study are included in the article/Supplementary Material. Further inquiries can be directed to the corresponding author.

## Supplementary Materials

The following supporting information can be downloaded at: https://www.mdpi.com/article/10.3390/nu18101611/s1, Figure S1: Antibiotic treatment significantly reduces gut microbial alpha diversity. Table S1: Inclusion and exclusion criteria for donor (toddlers aged 1–3 years). Table S2: Scoring standards for the disease activity index. Table S3: Histological scores of colon damage. Table S4: QPCR primers used in this work.
