# Supplementary material for: Fecal Microbiota Transplantation from Toddler Donors Ameliorated DSS-Induced Colitis in Mice by Reshaping Gut Microbiota
Source: Nutrients. 2026 May 19;18(10):1611. doi: 10.3390/nu18101611 (PMC13209430; doi:10.3390/nu18101611)
Supplement: Supplementary file 1 [file nutrients-18-01611-s001.zip › nutrients-4288872-supplementary.pdf]

Supplementary Materials

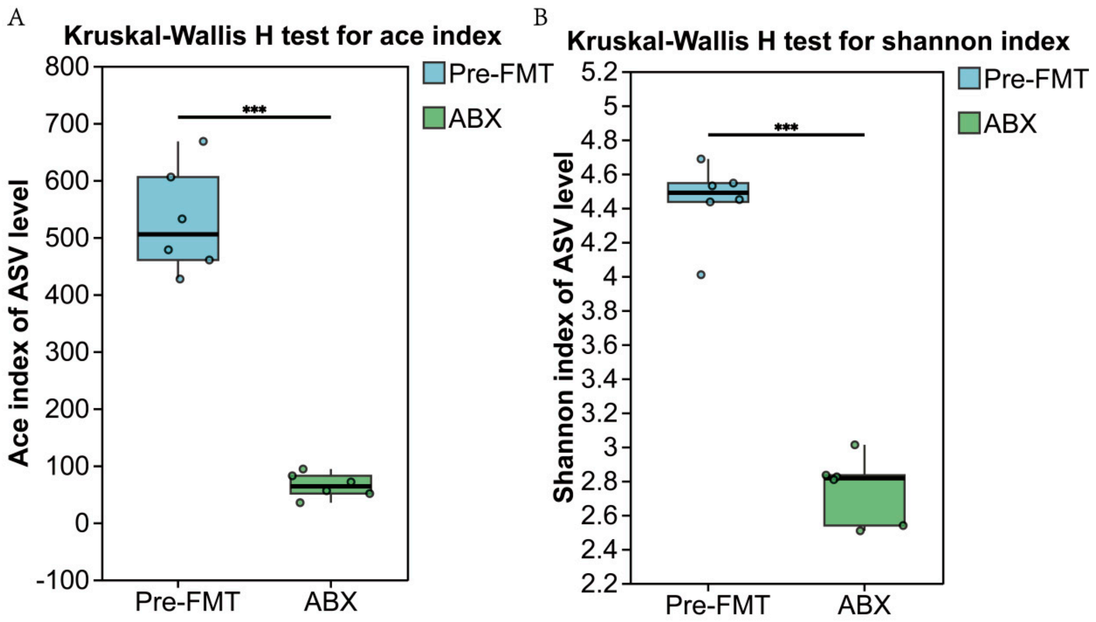

Figure S1. Antibiotic treatment significantly reduces gut microbial alpha diversity. (A) Comparison of ACE index between the Pre-FMT group and the ABX-treated group. The Kruskal-Wallis H test revealed a statistically significant decrease in ACE index following antibiotic treatment (***$p < 0.001$). (B) Comparison of Shannon index between the Pre-FMT group and the ABX-treated group. The Kruskal-Wallis H test demonstrated a significant reduction in Shannon diversity index in the ABX group compared to the control group (***$p < 0.001$).

Table S1. Inclusion and exclusion criteria for donor (toddlers aged 1–3 years).

Category	Inclusion Criteria	Exclusion Criteria
**Demographics**	• Age: 1–3 years<br>• Sex: male or female	• Age < 1 year or > 3 years
**Perinatal history**	• Term delivery (≥ 37 weeks of gestation)<br>• Normal birth weight (2.5–4.0 kg)<br>• Breastfed for at least 6 months	• Preterm delivery (< 37 weeks)<br>• Low birth weight (< 2.5 kg) or macrosomia (> 4.0 kg)<br>• Formula-fed exclusively without breastfeeding

Category	Inclusion Criteria	Exclusion Criteria
**Medical history**	• No history of gastrointestinal diseases (e.g., chronic diarrhea, constipation, inflammatory bowel disease)<br>• No history of allergic diseases (e.g., atopic dermatitis, food allergy)<br>• No history of genetic, metabolic, or immunodeficiency disorders<br>• No history of autoimmune diseases<br>• No history of known or suspected infectious disease	• History of gastrointestinal disorders<br>• History of allergic diseases<br>• History of genetic, metabolic, or immunodeficiency disorders<br>• History of autoimmune diseases<br>• History of known or suspected infectious disease
**Medication use**	• No antibiotic use in the past 6 months<br>• No probiotic or prebiotic use in the past 6 months	• Antibiotic use within 6 months prior to fecal collection<br>• Probiotic or prebiotic use within 6 months prior to fecal collection
**Growth and development**	• Normal growth and development according to WHO growth standards	• Growth retardation or developmental delay
**Stool quality**	• Stool samples collected within 2 hours of defecation and stored at –80°C within 30 minutes	• Stool samples not processed promptly or improperly stored

Table S2. Scoring standards for the disease activity index.

Score	Weight loss (%)	Stool consistency	Fecal blood content[a]
0	None	Granular hard stools	No stool blood
1	1–5%	Soft stools, granular shapes	Color change from light green to green within 10 s
2	6–10%	Very soft, shapeless stools	Color change from green to blue within 30 s
3	11–18%	Watery stools (Diarrhea)	Color change from green to dark blue immediately
4	> 18%		Visible rectal bleeding

[a] The hemoccult positive degree was assessed by using the Fecal Occult Blood Test Kit (Brybio, Beijing, China) based on color change.

Table S3. Histological scores of colon damage.

Score	Inflammation	Extent	Crypt damage
0	None	None	Intact crypt, no damage
1	Slight	Mucosa	Basal 1/3 crypt damaged
2	Moderate	Mucosa and Submucosa	Basal 2/3 crypt damaged
3	Severe	Transmural colonic tissues	Crypt lost but surface epithelium present
4			Crypt and surface epithelium lost

Table S4. QPCR primers used in this work.

Gene	Forward primer(5’-3’)	Reverse primer (5’-3’)
β-actin	GTGACGTTGACATCCGTAAAGA	GCCGGACTCATCGTACTCC
MUC2	ATGCCCACCTCCTCAAAGAC	GTAGTTTCCGTTGGAACAGTGAA
ZO-1	GCTTTAGCGAACAGAAGGAGC	TTCATTTTTCCGAGACTTCACCA
Occludin	TTGAAAGTCCACCTCCTTACAGA	CCGGATAAAAAGAGTACGCTGG

Claudin-15	ATGTCGGTAGCTGTGGAGAC	GGACGGAAAGTCCCAGCAG
IL-6	CCAAGAGGTGAGTGCTTCCC	CTGTTGTTCAGACTCTCTCCCT
Tnf-α	CCTGTAGCCCACGTCGTAG	GGGAGTAGACAAGGTACAACCC
IL-1β	GCAACTGTTCCTGAACTCAACT	ATCTTTTGGGGTCCGTCAACT
IL-18	GACTCTTGCGTCAACTTCAAGG	CAGGCTGTCTTTTGTCAACGA
NF-κB	ATGGCAGACGATGATCCCTAC	TGTTGACAGTGGTATTTCTGGTG
JAK1	CTCTCTGTCACAACCTCTTCGC	TTGGTAAAGTAGAACCTCATGCG
JAK2	TTGTGGTATTACGCCTGTGTATC	ATGCCTGGTTGACTCGTCTAT
STAT1	TCACAGTGGTTCGAGCTTCAG	GCAAACGAGACATCATAGGCA
STAT6	CTCTGTGGGGCCTAATTTCCA	CATCTGAACCGACCAGGAACT
